# The Contribution of Fluorine ^18^F-FDG PET/CT to Lung Cancer Diagnosis, Staging and Treatment Planning

**DOI:** 10.4274/mirt.53315

**Published:** 2018-06-07

**Authors:** Emine Budak, Gürsel Çok, Ayşegül Akgün

**Affiliations:** 1 University of Health Sciences, İzmir Dr. Suat Seren Chest Diseases and Surgery Training and Research Hospital, Clinic of Nuclear Medicine, İzmir, Turkey; 2 Ege University Faculty of Medicine, Department of Chest Diseases, İzmir, Turkey; 3 Ege University Faculty of Medicine, Department of Nuclear Medicine, İzmir, Turkey

**Keywords:** Lung cancer, positron emission tomography, survival analysis

## Abstract

**Objective:**

Lung cancer is the most common cause of cancer-related death throughout the world, and the correct choice of treatment based on early diagnosis and staging increases the chance of survival. The present study aims to investigate the contribution of fluorine 18-fluorodeoxyglucose-positron emission tomography/computed tomography (^18^F-FDG PET/CT) to the management of lung cancer.

**Methods:**

In this study, 50 patients who underwent ^18^F-FDG PET/CT for lung cancer diagnosis and staging between February 2012 and February 2014 were included. The maximum standardized uptake value (SUV_max_) of the primary lung lesion along with other findings of ^18^F-FDG PET/CT and the results of histopathologic and conventional examinations were evaluated retrospectively. The mean survival time of patients was determined, and the findings were compared by using statistical methods.

**Results:**

Histopathologic examinations revealed 51 lung cancers in 50 patients. The sensitivity, accuracy and positive predictive value of ^18^F-FDG PET/CT in detecting primary malignancy were 94%, 94%, 100%, respectively. Adenocarcinoma (n=23, 16.8±13.5) and squamous cell carcinoma (n=15, 17.9±5.6) did not differ significantly regarding their mean SUV_max_ values (p=0.2). A statistically significant positive correlation (r=0.4) was identified between tumor size and SUV_max_ value for 51 tumors (p=0.002). The ^18^F-FDG PET/CT result was true negative in nine, false positive in six, true positive in two, and false negative in four patients who underwent histopathologic evaluation of their lymph nodes. The ^18^F-FDG PET/CT changed treatment planning in 34% of the patients. No significant relationship was identified between SUV_max_ value of the tumor and patient survival in patients (p=0.118).

**Conclusion:**

The present study concluded that PET/CT was an efficient method in the diagnosis and staging of lung cancer since it provided useful information in addition to conventional methods. It was also observed that PET/CT scanning resulted in a change in therapeutic plans in the majority of patients. However, there was no statistically significant relationship between survival and the SUV_max_ of the primary mass.

## Abstract (tr)

**Amaç:**

Akciğer kanseri, tüm dünyada kansere bağlı ölümlerin en sık nedenidir. Erken tanı ve evrelemeye bağlı doğru tedavi seçimi akciğer kanserinde sağkalımı artıran bir faktördür. Amacımız akciğer kanseri yönetimine flor 18-fluorodeoksiglukoz-pozitron emisyon tomografisi/bilgisayarlı tomografinin (^18^F-FDG PET/BT) katkısını araştırmaktır.

**Yöntem:**

Çalışmamıza Şubat 2012-Şubat 2014 tarihleri arasında akciğer kanseri tanı ve evrelemesi amacıyla ^18^F-FDG PET/BT yapılan 50 hasta dahil edildi. ^18^F-FDG PET/BT’de akciğerde saptanan primer lezyonun maksimum standart tutulum değeri (SUV_maks_) ve diğer bulguları ile histopatolojik ve konvansiyonel yöntem sonuçları retrospektif olarak değerlendirildi. Hastaların ortalama sağkalım süresi belirlendi. İstatistiksel yöntemler kullanılarak bulgular karşılaştırıldı.

**Bulgular:**

Elli hastada histopatolojik olarak toplam 51 akciğer kanseri saptandı. ^18^F-FDG PET/BT’nin primer maligniteyi saptamada duyarlılık, doğruluk ve pozitif prediktif değeri sırasıyla %94, %94, %100 saptandı. Adenokarsinom (n=23, 16,8±13,5) ve skuamöz hücreli karsinom (n=15, 17,9±5,6) ortalama SUV_maks_ değeri açısından anlamlı bir farklılık göstermedi (p=0,2). Elli bir tümör için tümör boyutu ile SUV_maks_ değeri arasında istatistiksel olarak anlamlı pozitif korelasyon (r=0,4) mevcuttu (p=0,002). ^18^F-FDG PET/BT, histopatolojik olarak lenf nodu değerlendirilen hastaların; dokuzunda gerçek negatif, altısında yanlış pozitif, ikisinde gerçek pozitif ve dördünde yanlış negatifti. ^18^F-FDG PET/BT, hastaların %34’ünde tedavi planını değiştirdi. Tümörün SUV_maks_ değeri ile hasta sağkalımı arasında anlamlı bir ilişki yoktu (p=0,118).

**Sonuç:**

Çalışmamızda PET/BT’nin konvansiyonel yöntemlere ek önemli bilgiler sağlayarak akciğer kanseri tanı ve evrelemesinde etkin bir yöntem olduğu sonucuna varıldı. Ayrıca PET/BT’nin evrelemeye katkısıyla hastaların önemli bir kısmında tedavi planında değişikliğe neden olduğu görüldü. Ancak primer kitlenin SUV_maks_ değeri ile hastaların sağkalımı arasında istatistiksel olarak anlamlı ilişki saptanmadı.

## Introduction

Lung cancer is the most frequent malignancy throughout the world since 1985 (1) and the leading cause of cancer-related death in both men and women (2). Curative treatment can be offered to lung cancer patients with early diagnosis, in whom computed tomography (CT) is the first diagnostic tool (3). One of the most important prognostic factors in lung cancer is tumor stage. Hence, proper staging is very important when determining prognosis and choosing appropriate treatment. T-staging is based on CT findings, although this modality may be unable to distinguish a tumor from atelectasis or show disease extent. Fluorine 18-fluorodeoxyglucose positron emission tomography/CT (^18^F-FDG PET/CT) is an effective method for the diagnosis, staging, evaluation of treatment response, follow-up for recurrence and re-staging of lung cancer. In our retrospective study, we investigated the contribution of ^18^F-FDG PET/CT to lung cancer diagnosis, staging and management, as well as the prognostic and survival effects of the maximum standardized uptake value (SUV_max_) of the primary lesion in an ^18^F-FDG PET/CT.

## Materials and Methods

The present study included 50 patients who have been referred to Ege University Faculty of Medicine, Department of Nuclear Medicine between February 2012 and February 2014, and who have undergone ^18^F-FDG PET/CT scanning for the diagnosing and staging of lung cancer. Patients were injected intravenously with 250-400 MBq of ^18^F-FDG at least four hours after fasting for ^18^F-FDG PET/CT imaging. Approximately one hour after the tracer injection, PET and CT scans were obtained using a PET-CT scanner (biograph high-definition 16-slice CT, Siemens Healthcare, Erlangen, Germany), and the PET and CT images were loaded onto three-dimensional workstations. Visual and semi-quantitative evaluation of the lesions observed on ^18^F-FDG PET/CT were carried out. In case of non-physiologically enhanced areas of activity associated with soft/bone tissue on CT, the finding was identified as “PET lesion”, and these PET lesions were interpreted as malignant if either the SUV_max_ value >2.5 or when the FDG uptake was significantly higher than the background tissue. Adrenal gland lesions with a higher FDG uptake than the liver were considered as malignant. A total of 30 patients or their close relatives were accessed to assess survival. One of those 30 patients had synchronous double primary lung cancers. The correlation between the SUV_max_ value of the primary lesion and patient survival was evaluated. The findings of ^18^F-FDG PET/CT were compared with radiologic and histopathologic results as well as the final clinical decisions and the determined stages.

### Statistical Analysis

The IBM SPSS V 21.0 software was used for statistical analysis. Kolmogorov-Smirnov and Shapiro-Wilk (S-W) tests were utilized for normality analysis of data distribution, and Mann-Whitney U test was applied to determine the differences between the two groups in case of non-normal distribution. A Spearman’s rho correlation test was utilized to investigate the correlation between the parameters, and the Kaplan Meier method was used for survival analysis. The differences in survival curves taking different variables into account were tested by using the log-rank test.

## Results

This study included 50 patients (46 males, four female, mean age 63.0±8.6) who underwent ^18^F-FDG PET/CT. A histopathologic diagnosis of lung cancer was obtained in all patients. There were 51 tumors in 50 patients since one patient had synchronous double primary lung cancers that have been diagnosed as adenocarcinoma. The histopathologic results of the lung lesions revealed 23 adenocarcinomas, 15 squamous cell carcinomas (SCC), four small-cell lung cancers (SCLC), two large-cell neuroendocrine carcinomas, one adenoid cystic carcinoma, one carcinoid tumor and five non-SCLC ​[(NSCLC)​, with no specific classifications]. Patient and tumor characteristics are summarized in Table 1. In terms of detecting malignancy, the ^18^F-FDG PET/CT yielded true positive results in 48 and false negative findings in three cases out of the 51 tumors. The tumor size and histopathologic features of these three false negative lesions were 0.6 cm adenocarcinoma (SUV_max_: 1.4), 1.3 cm adenocarcinoma (SUV_max_: 1.6) and 1 cm carcinoid tumor (SUV_max_: 0.8). In the patient who had synchronous double primary lung cancers, the adenocarcinoma of 0.6 cm (SUV_max_: 1.4) had a false negative finding whereas the 2.7 cm-sized (SUV_max_: 18.6) adenocarcinoma was detected. The sensitivity, accuracy and positive predictive value of ^18^F-FDG PET/CT in detecting primary malignancy were calculated as 94%, 94% and 100%, respectively. The mean SUV_max_ values of adenocarcinoma (n=23) and SCC (n=15) lesions was 16.8±13.5 and 17.9±5.6, respectively. These two types of lung cancer were similar in terms of SUV_max_ values (p=0.2).

**Figure 1:**
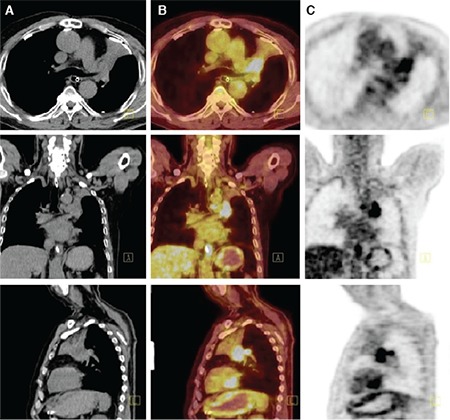
Axial, coronal, and sagittal CT images (A), fused PET/CT images (B) and PET images (C) of the patient with small cell lung carcinoma. The tumor size could not clearly be assessed on CT images. The fused PET/CT images enables differentiation of tumor and atelectasis areas due to intense FDG uptake of the tumor area [tumor maximum standardized uptake value (SUV_max_): 10, atelectasis SUV_max_: 3.7], thus, the tumor size has been correctly calculated

**Figure 2:**
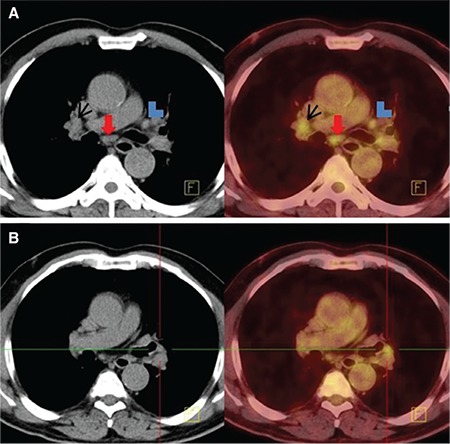
A and B demonstrate axial sections of CT and fused PET/CT images in the patient with lung adenocarcinoma. Right hilar [black arrow in A, maximum standardized uptake value (SUV_max_): 4.1], subcarinal (red arrow in A, SUV_max_: 4.2) and left interlobar hypermetabolic lymph nodes (in B, SUV_max_: 3.8) seen on fused PET/CT were evaluated as anthracosis using histopathologic sampling. The hypermetabolic subaortic lymph node (blue arrowhead in A, SUV_max_: 4.1) was histopathologically reactive

The correlation between tumor diameter and SUV_max_ was evaluated for all tumors. The mean tumor diameter of the 51 tumors was 5.0±2.9 cm. Tumor sizes ranged from 0.6 cm to 12.3 cm. A statistically significant positive correlation (r=0.4) was identified between tumor size and SUV_max_ (p=0.002). Two patients had post-obstructive atelectasis due to the tumor, and the ^18^F-FDG PET/CT was able to distinguish between the tumor and the atelectasis owing to an intense FDG uptake in the tumor area. Thus, the tumor size was correctly calculated (Figure 1). The histopathologic results of the lymph nodes were compared with nodal staging according to ^18^F-FDG PET/CT. Histopathologic evaluation of the lymph nodes was performed on 21 patients. The ^18^F-FDG PET/CT findings were interpreted as true negative in nine, false positive in six, true positive in two and false negative in four patients. In this regard, 11/21 (52%) of the patients were correctly staged by ^18^F-FDG PET/CT, while 4/21 (19%) patients were incorrectly down-staged and 6/21 (28%) were incorrectly up-staged by PET-CT. Within the group of 15 histopathologically N0 patients, the ^18^F-FDG PET/CT was N0 in nine (true negative) while it was false positive in the remaining six patients. ^18^F-FDG PET/CT identified subcarinal, right hilar and left interlobar hypermetabolic lymph nodes in one of the six patients with a false positive result (Figure 2). Thus, the patient was staged as N3 on ^18^F-FDG PET/CT. Histopathologically, these lymph nodes were assessed as anthracosis and the patient was down-staged. When assessed on a patient-by-patient basis, hilar and interlobar lymph nodes were most commonly recorded as false-positives on ^18^F-FDG PET/CT (in 66% of patients with false-positive lymph nodes). A total of 20 distant metastatic sites (eight bone, five liver, three surrenal gland, two brain, one spinal, one pons metastases) were detected in 14 patients (28% of all patients) by ^18^F-FDG PET/CT . Of these metastases, 12/20 (60%) could only be detected by ^18^F-FDG PET/CT. When evaluated on a patient-by-patient basis, in 14% (7/50) of all patients, distant metastases were detected only by ^18^F-FDG PET/CT. 

**Figure 3:**
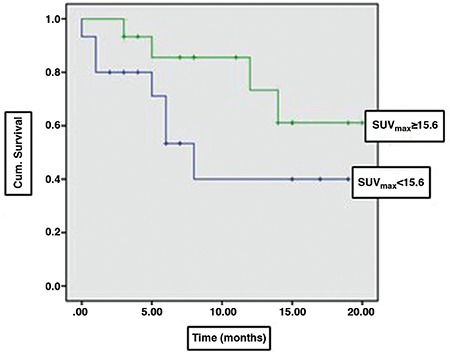
Survival curves of patients according to their maximum standardized uptake value (SUV_max_) values; there was no significant difference in terms of mean survival time between SUV_max_ values below 15.6 (10.3±2.2 months) and those above 15.6 (15.9±1.6 months) (p=0.118)

^18^F-FDG PET/CT led to a change in treatment plan based on conventional methods in 17/50 (34%) patients. Through conventional staging, 11 of these 17 patients were classified as “at a potentially operable” stage (stage 1-3A), five were classified as “at a potentially inoperable” stage (stage 3B-4), and one patient had a lesion that was assessed to be benign by conventional methods. Within the group of 11 patients classified as “at a potentially operable” stage, four had N3 lymph node metastases and five had distant metastases as identified by ^18^F-FDG PET/CT. As a result, these patients underwent chemotherapy thus avoiding unnecessary surgery in 9/50 (18%) patients. In the other two patients, N1 lymph node metastasis was detected by ^18^F-FDG PET/CT, and neoadjuvant chemotherapy was started prior to the operation. Within the group of five patients who were classified as “at a potentially inoperable” stage, three were identified with M1 disease by conventional staging. The areas considered as distant metastases in these three patients were interpreted as benign by ^18^F-FDG PET/CT and thus were accepted as candidates for curative treatment. The other two patients were categorized as stage 3B by conventional methods and were thus planned for chemotherapy and localized radiotherapy. However, distant metastases were detected by ^18^F-FDG PET/CT in these two patients who underwent chemotherapy and palliative radiotherapy. Another patient had a lesion that was interpreted as benign by conventional methods while the ^18^F-FDG PET/CT evaluated this mass as malignant, and the patient was started on chemotherapy. The impact of ^18^F-FDG PET/CT on patient management is listed in Table 1. Finally, the correlation between SUV_max_ value of the primary lesion and patient survival was evaluated in 30 patients, one of whom was the patient who had synchronous double primary lung cancers. Primarily, the median SUV_max_ (median SUV_max_= 15.6) was calculated and accepted as the “cut-off” value. The mean survival time was 10.3±2.2 months in patients with SUV_max_ <15.6, and 15.9±1.6 months in those with SUV_max_ ≥15.6. There was no significant difference between the two groups in terms of survival (p=0.118) (Figure 3).

## Discussion

Lung cancer is the most common cause of cancer-related death worldwide, and early diagnosis is important for the application of curative treatments. ^18^F-FDG PET/CT is used commonly to differentiate benign and malignant lung lesions. Gupta et al. (4) reported the sensitivity and specificity of ^18^F-FDG PET/CT in benign-malignant distinction of indeterminate solitary pulmonary nodules as 93% and 88%, respectively. ^18^F-FDG PET/CT might yield a false positive result in inflammatory conditions, tuberculosis or granulomatous lesions. A false negative ^18^F-FDG PET/CT result is generally associated with nodules measuring less than 1 cm, carcinoid tumors or bronchoalveolar carcinomas (BAC) (5,6,7,8,9). In our study, ^18^F-FDG PET/CT provided a true positive result in 94% and a false negative result in 6% of 51 lesions. The tumors with a false negative ^18^F-FDG PET/CT included two adenocarcinomas (0.6 cm and 1.3 cm) and one carcinoid tumor (1 cm). The small size and presence of a carcinoid tumor may have contributed to the false negative results. In the present study, the sensitivity, accuracy and positive predictive value of ^18^F-FDG PET/CT in the detection of primary malignancy were calculated as 94%, 94% and 100%, respectively. These results are consistent with that reported in the literature. Several studies indicated that the mean SUV and SUV_max_ differ according to histologic subtypes of lung cancer. In particular, it has been reported that the SUV_max_ of carcinoid tumors and BAC are low (10,11). Vesselle et al. (12) found that the SUV_max_ of BAC was lower than all other subtypes, and that non-BAC adenocarcinomas had lower SUV_max_ values than SCC. The majority of studies in the literature report that the SUV_max_ of adenocarcinomas is lower than that of SCCs (13,14). However, in our study, no statistically significant difference was identified between adenocarcinoma (16.8±13.5) and SCC (17.9±5.6) in terms of mean SUV_max_ (p=0.2). Several studies showed that tumor size is a prognostic factor for survival in NSCLC (15). The correlation between tumor size and SUV_max_ has been previously assessed (16,17). Zhu et al. (18) reported a moderate positive correlation between SUV_max_ value and tumor size (r=0.642, p<0.001). In our study, a positive correlation (p=0.002) was identified between tumor size and SUV_max_, in line with findings of the earlier studies.

The detection of both lymph node and distant metastases is essential for proper staging in lung cancer. The sensitivity of ^18^F-FDG PET/CT in lymph nodes greater than 1 cm is high, although the accuracy and specificity rates are low (19). In a study by Detterbeck et al. (20), the false positive rate of PET in mediastinal lymph nodes was reported to be 13-22%, and the false negative rate as 5-8%. In our study, histopathologic lymph node evaluation was carried out in 21 of the 50 patients, and 52% of the 21 patients were correctly staged by ^18^F-FDG PET/CT whereas the rate of patients incorrectly down-staged or up-staged by ^18^F-FDG PET/CT were detected as 19% and 28%, respectively. In our study, the false positive and false negative lymph node rates in ^18^F-FDG PET/CT were found to be higher than that reported in the literature. This finding may be attributed to the fact that histopathologic evaluation was not performed in all lymph nodes evaluated as either positive or negative by ^18^F-FDG PET/CT. Furthermore; sarcoidosis, amyloidosis, anthracosis, tuberculosis and organized pneumonia may cause false positive results in lymph nodes. In one patient with a false positive lymph node on ^18^F-FDG PET/CT, histopathologic sampling revealed anthracosis. In addition, pulmonary infections or granulomatous diseases such as tuberculosis or sarcoidosis that are frequent in our country might have contributed to these outcomes. Further investigation of lymph node and distant organ metastases, particularly in patients being considered for surgical therapy, could prevent unnecessary surgery. Van Tinteren et al. (21) reported that performing an ^18^F-FDG PET/CT in addition to conventional methods as part of preoperative staging of NSCLC might avoid unwarranted surgery in one-fifth of patients. In a study on patients with NSCLC, an ^18^F-FDG PET/CT led to a change in disease stage as determined by conventional methods in 50.6% (41.1% up-staged, 9.5% down-staged) of patients, and alteration in treatment planning in 42.3% (22). In our study, the ^18^F-FDG PET/CT resulted in a change in the treatment plan as decided by conventional methods in 34% of all patients, and unnecessary surgery was prevented in 18% of the patients. In 6% of the patients, the ^18^F-FDG PET/CT was interpreted as negative for areas identified as metastasis through conventional methods, leading patients to possible curative therapy. In various studies, SUV_max_ of the primary tumor detected by ^18^F-FDG PET/CT was used to predict lung cancer prognosis and assess patient survival (23,24). Ahuja et al. (25) found that patients with primary tumors showing a high FDG uptake had lower survival rates than those with a low uptake, while Hoang et al. (26) found no significant association between FDG uptake of the primary tumor and survival in patients with advanced NSCLC. In a meta-analysis published by IASLC in 2008, the SUV_max_ of the primary tumor was a strong determinant of prognosis in NSCLC. However, the need to support this finding with multivariate analysis was emphasized (27). The present study evaluated the relationship between SUV_max_ of the primary tumor and patient survival and no significant difference was identified between those with SUV_max_ below 15.6 (10.3±2.2 months) and above 15.6 (15.9±1.6 months) in terms of mean survival time (p=0.118). Several previous studies support our findings, although a larger number of authors argue that SUV_max_ could be used to predict prognosis and assess survival in case of lung cancer. Our results might be related to the limited number of patients in the study as well as the differences between the two groups (e.g. age, genetic factors, stage, tumor pathology).

## Conclusion

Despite the limitations of our study, such as limited number of patients, most of the results are consistent with previous studies. In our study, it is concluded that ^18^F-FDG PET/CT contributes to both the diagnosis and management of lung cancer by providing valuable information in addition to conventional methods.

## Figures and Tables

**Table 1:**
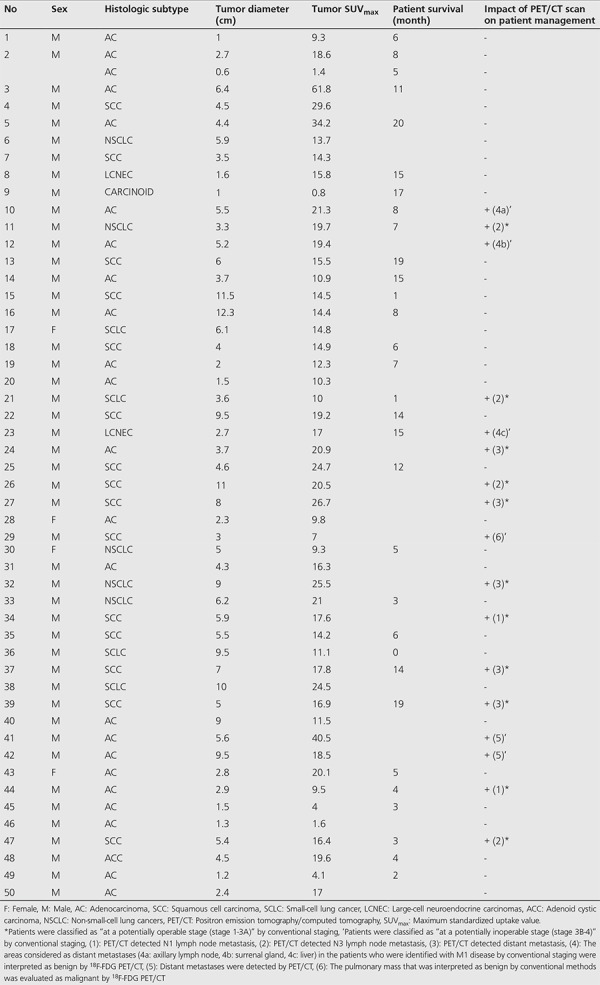
Patient-tumor characteristics and the impact of positron emission tomography/computed tomography on patient management
